# ANALYSIS OF SURGICAL SITE INFECTIONS IN PEDIATRIC PATIENTS AFTER ORTHOPEDIC SURGERY: A CASE-CONTROL STUDY

**DOI:** 10.1590/1984-0462/;2017;35;1;00011

**Published:** 2017

**Authors:** Mariana de Queiroz Leite Chagas, Ana Maria Magalhães Costa, Pedro Henrique Barros Mendes, Saint Clair Gomes

**Affiliations:** a Instituto Nacional de Traumatologia e Ortopedia Jamil Haddad - Ministério da Saúde, Rio de Janeiro, RJ, Brasil.; b Instituto Nacional de Saúde da Mulher, da Criança e do Adolescente Fernandes Figueira - Fundação Oswaldo Cruz - Ministério da Saúde, Rio de Janeiro, RJ, Brasil.

**Keywords:** Surgical wound infection, Orthopedics, Risk factors, Nosocomial infections, Child

## Abstract

**Objectives:**

To describe the rate of surgical site infections in children undergoing orthopedic surgery in centers of excellence and analyze the patients’ profiles.

**Methods:**

Medical records of pediatric patients undergoing orthopedic surgery in the Jamil Haddad National Institute of Traumatology and Orthopedics from January 2012 to December 2013 were analyzed and monitored for one year. Patients diagnosed with surgical site infection were matched with patients without infection by age, date of admission, field of orthopedic surgery and type of surgical procedure. Patient, surgical and follow-up variables were examined. Descriptive, bivariate and correspondence analyses were performed to evaluate the patients’ profiles.

**Results:**

347 surgeries and 10 surgical site infections (2.88%) were identified. There was association of infections with age - odds ratio (OR) 11.5 (confidence interval - 95%CI 1.41-94.9) -, implant - OR 7.3 (95%CI 1.46-36.3) -, preoperative period - OR 9.8 (95%CI 1.83-53.0), and length of hospitalization - OR 20.6 (95%CI 3.7-114.2). The correspondence analysis correlated the infection and preoperative period, weight, weight Z-score, age, implant, type of surgical procedure, and length of hospitalization. Average time to diagnosis of infection occurred 26.5±111.46 days after surgery.

**Conclusions:**

The rate of surgical site infection was 2.88%, while higher in children over 24 months of age who underwent surgical implant procedures and had longer preoperative periods and lengths of hospitalization. This study identified variables for the epidemiological surveillance of these events in children. Available databases and appropriate analysis methods are essential to monitor and improve the quality of care offered to the pediatric population.

## RESUMO

**Objetivos:**

Descrever taxa de infecção no sítio cirúrgico em crianças submetidas à cirurgia ortopédica em centro de referência e analisar o perfil desses pacientes.

**Métodos:**

Verificados prontuários de pacientes pediátricos submetidos à cirurgia ortopédica de janeiro de 2012 a dezembro de 2013 no Instituto Nacional de Traumatologia e Ortopedia Jamil Haddad com um ano de seguimento. Pacientes com diagnóstico de infecção de sítio cirúrgico foram pareados com pacientes sem esse diagnóstico com base na idade, na data de internação, na área de atuação ortopédica e no tipo de procedimento cirúrgico. Averiguadas variáveis do paciente, da cirurgia e do seguimento. Realizadas análises descritivas, bivariadas e de correspondência para avaliação do perfil dos pacientes.

**Resultados:**

Identificadas 347 cirurgias e 10 infecções de sítio cirúrgico (2,88%). Houve associação de infecção com idade Odds Ratio (OR) 11,5 (intervalo de confiança - IC95% 1,41-94,9), implante OR 7,3 (IC95% 1,46-36,3), tempo pré-operatório OR 9,8 (IC95% 1,83-53,0) e tempo de internação OR 20,6 (IC95% 3,7-114,2). A análise de correspondência correlacionou a infecção com tempo pré-operatório, peso, escore Z de peso, idade, implante, tipo de procedimento e tempo de internação. O tempo médio de diagnóstico da infecção foi de 26,5±111,46 dias após cirurgia.

**Conclusões:**

A taxa de infecção no sítio cirúrgico foi de 2,88%, maior em crianças acima de 24 meses, que realizaram implante, tiveram mais tempo pré-operatório e de internação. Este estudo identificou variáveis de vigilância epidemiológica desses eventos em crianças. Bancos de dados e métodos de análises adequados são fundamentais para o acompanhamento e o aprimoramento do cuidado ofertado à população pediátrica.

## INTRODUCTION

Surgical site infections (SSIs) are severe events that have direct repercussions on patients’ surgical morbidity and mortality, generating both direct and indirect costs for the health care system, society, and their families.^1^ ^,^ ^2^ These events are directly related to the increased hospitalization periods and a greater number of diagnostic and therapeutic procedures.^3^

SSI control is an important indicator for monitoring surgical patients for administrators and health care professionals.^4^ ^,^ ^5^ The systematic monitoring of this rate is conducive to identifying groups at greater risk and following up these events, which helps plan preventive actions and develop strategies to control these infections.^6^ ^,^ ^7^

The adult population has an estimated SSI rate of approximately 11%, which is related to the clinical and epidemiological characteristics of this population and the structural characteristics of the studied sites.^8^ The SSI rate for the pediatric population varies between 2.5 and 20.0%.^9^ ^,^ ^10^ The variability in the SSI rate in the pediatric population may be due to differences between hospitals, procedures, follow-up, and intrinsic factors related to pediatric patients.^11^ ^,^ ^12^ Increased SSI estimation accuracy for the pediatric population involves using prerequisites as specific protocols for SSI data collection and referring patients to centers of excellence for pediatric care, combining expertise and case studies.

The objectives of this study were to describe the SSI rate in children undergoing orthopedic surgery in a center of excellence and analyze the profiles of those patients.

## METHOD

This study was conducted in the pediatric ward of the Jamil Haddad National Institute of Traumatology and Orthopedics (INTO), a Ministry of Health hospital; its excellence is a national reference of the Brazilian Unified Health System (SUS) for the treatment of osteoarticular diseases and rehabilitation.

Medical records of pediatric patients undergoing orthopedic surgery from January 2012 to December 2013 were analyzed. All patients who had a follow-up record of up to one year after the date of the procedure and met the “Surgical patient subject to routine epidemiological surveillance” criteria, according to the *Guidelines and Diagnostic Criteria for Infection Related to Health Care* by the Brazilian Health Surveillance Agency/Ministry of Health (Anvisa/MS), were included in the study.^13^ ^,^ ^14^

Patients whose medical records showed a SSI diagnosis were matched with patients without SSI, considering the following variables: age, date of admission, orthopedic field, and type of surgical procedure.

Of the INTO’s 12 orthopedic fields, only the pediatric and spinal surgical specialties were analyzed. The type of surgical procedure was determined according to the SUS procedure table.^12^ All patients whose records presented potentially contaminated, contaminated, or infected conditions in the preoperative period were excluded from the analysis, according to the Center for Disease Control and Prevention (CDC).^15^

Data were registered in a standard form subdivided into three sections with the following information:

- Patient: sex (male/female); age (≤ 24 months/>24 months); weight (<50^th^ percentile/≥50^th^ percentile - P50); weight Z-score (<-1, >-1 and <1, >1), calculated using the Anthro software, by the World Health Organization (WHO);^16^ presence of underlying disease; the American Society of Anesthesiologists (ASA) Physical Status Classification;^17^ ^,^ ^18^ and orthopedic surgery field (pediatric/spine).
- Surgical treatment: preoperative hospitalization period, that is, the difference between the date of the surgical procedure and the date of admission; total period of hospitalization, that is, the difference between the discharge date and the date of admission; duration of the surgery, classified as ≤120 minutes and >120 minutes; use of orthopedic implants (yes or no); and type of orthopedic surgical procedure (lower limbs/other parts), according to the SUS procedure table.^12^
- Follow-up: number of outpatient visits in the post-discharge monitoring period; time to diagnosis of SSI (difference between the date of SSI detection and the date of surgery); type of SSI (superficial, deep, or organ/space);^13^ ^,^ ^17^ material collection for microbiological analysis; and identification of the etiologic agent.

The SSI rate was estimated based on descriptive analyses. The patients’ profiles were examined considering bivariate and correspondence analyses. The odds ratio (OR) was used to assess the strength of association between exposure (patient and surgical treatment variables) and outcome (SSI event) with a 95% confidence interval (95%CI). Fisher’s and chi-squared tests were applied to test statistically significant associations with a 0.05 significance level. The multiple correspondence analysis (MCA) was applied to enable the graphical visualization of the correlation between variable categories of exposure and outcome. In general, categories in the same quadrant on the chart indicate greater association.

The Epi Info™ software, Version 7.0, was used to create the database, and the Statistical Package for the Social Sciences (SPSS) software, Version 21, was used to perform statistical analyses. This study was approved by the INTO Research Ethics Committee (REC), Process No. 45324715.6.0000.5273, on May 21, 2015.

## RESULTS

**Figure 1:**
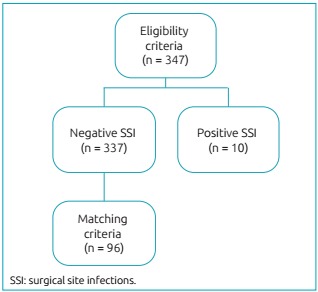
Flow chart of medical records selection of patients who underwent orthopedic surgery in the Jamil Haddad National Institute of Traumatology and Orthopedics, Rio de Janeiro, 2012-2013.

Researchers identified 347 medical records that met the criteria for inclusion. Of these, 10 (2.88%) had a SSI record of up to one year after the surgical procedure. Using the matching criteria, we selected 96 patients (Fig. 1).

The profile of the total sample was characterized by a higher proportion of boys (56%), younger than 24 months of age (51.9%), weighing below P50 (53.8%), with weight Z-score ranging between -1 and 1 (53.8), and predominantly of Class I ASA Classification (61.3%). For the most part, patients did not suffer from any underlying diseases (69.8%). Among the practice fields analyzed, only one patient fit into the spinal surgical specialty category (Table 1). For this set of variables, we did not identify statistically significant differences between the SSI group and the control groups, except for the age with 11.6 OR (95%CI 1.41-94.96) for the group of children over 24 months of age (Table 1).

**Table 1:**
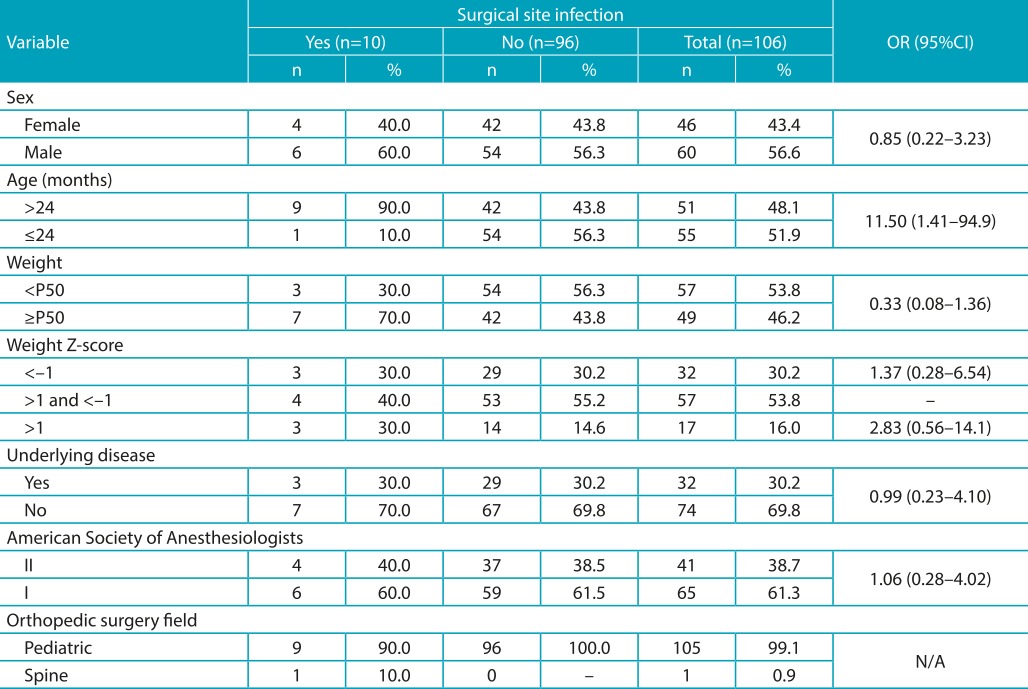
Demographic characteristics of patients submitted to orthopedic surgery.

OR: odds ratio; 95% CI: 95% Confidence Interval; N/A: not applicable.

Regarding the surgical treatment data, we established that 60.4% of patients did not have implant records, 74.5% underwent lower limb surgery, 93.4% had a preoperative period of up to one day, 92.5% had a surgery that lasted less than or equal to 120 minutes, and 93.4% presented a length of hospitalization of up to five days (Table 2). We identified statistically significant differences for the incidence of SSI for the following variables: implant (OR=7.3; 95%CI 1.46-36.3), length of hospitalization (OR=20.6; 95%CI 3.7-114.2), and preoperative period (OR=9.8; 95%CI 1.83-53.0).

**Table 2:**
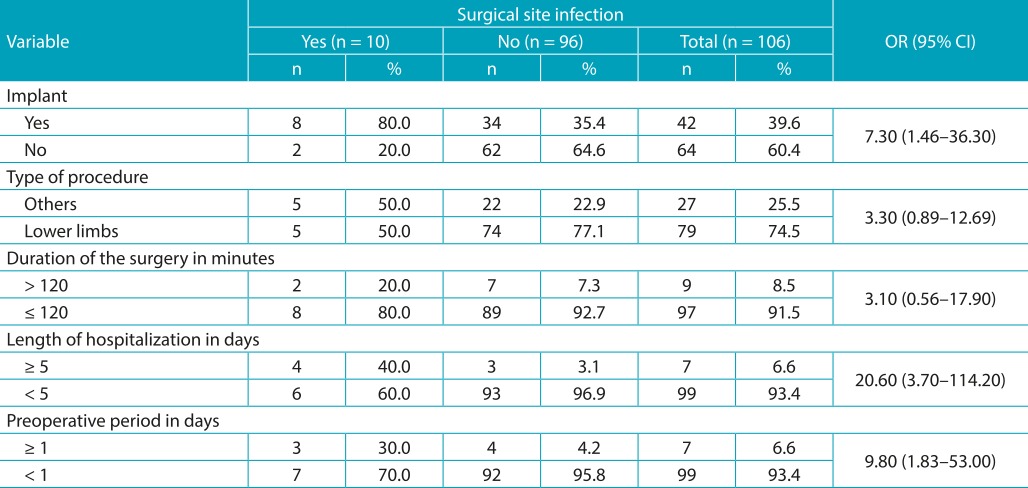
Characteristics of the surgeries in patients submitted to orthopedic surgery.

OR: odds ratio; CI: 95% Confidence Interval. The corresponding values are Positive SSI - N = 2 and 8 (20 and 80%) // Negative SSI - N = 7 and 89 (6.0 and 84%), without OR and 95% CI alterations.

**Chart 1:**
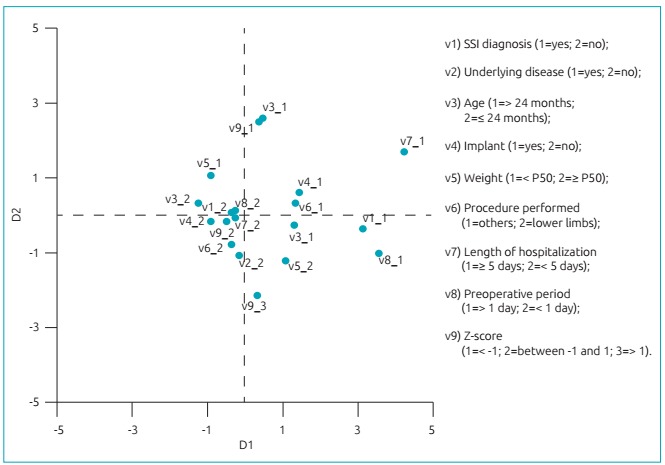
Multiple correspondence analysis of the incidence of surgical site infections, considering patient and surgical treatment variables, in the Jamil Haddad National Institute of Traumatology and Orthopedics, Rio de Janeiro, 2012-2013.

The MCA indicates (Chart 1) that the SSI diagnosis category (V1-1), located on the chart’s second quadrant, is related to categories with respect to age over 24 months (V3-1), weight greater than or equal to P50 (V5-2), weight Z-score higher than 1 (V9-3), and preoperative period longer than one day (V8-1), indicating a correlation between these variables.

During the patients’ follow-up period, we found eight superficial SSIs (80%) and two organ/space SSIs (20%). Seven patients (70%) had a collection record of biological material from the infected area for microbiological analysis; the etiologic agent was confirmed in five patients (50%). *Staphylococcus aureus* was the etiologic agent found in four patients, one of which had a methicillin-resistant *Staphylococcus aureus* (MRSA) profile. Only one case was isolated, which was the *Enterobacter cloacae* etiologic agent. Patients with SSI had an average of five outpatient visits within a year. Average time to diagnosis of SSI was 26.5 days (5 to 343 days).

## DISCUSSION

This study was motivated by the importance of gathering information on SSIs for the pediatric population. The emphasis on orthopedic surgery was due to the complexity of these procedures and their increased volume in the health care system, which supports the need for data to provide appropriate epidemiological surveillance.^4^ ^,^ ^7^ ^,^ ^19^ This study establishes that the SSI rate was 2.88%, which is compatible with the parameters reported in the research found for the adult population. During the study period, the rate found for all types of infection ranged from 3.40 to 6.61%.

The site chosen to conduct the study was appropriate because orthopedic surgeries generally occur in a smaller proportion of the pediatric population, reflecting the difficulty to select cases. Since INTO is a center of excellence for this type of treatment, it concentrates cases that are more complex and vulnerable to the development of the infection. Local characteristics may have had an influence on the estimated rate. However, even within this low-frequency scenario, the study confirmed that patient, surgical, and follow-up variables are related to SSI.

For the set of patient variables, age indicated a statistically significant association (OR=11.6 for patients over 24 months of age). A possible explanation for this association may be the fact that older patients tend to have more complex procedures performed due to musculoskeletal maturity.^10^ ^,^ ^19^ ^,^ ^20^ ^,^ ^21^

Regarding surgical treatment data, the use of implants indicated a risk of SSI development (OR=7.3). Several authors state that the presence of an implant is a risk factor for infection owing to the predisposition to bacterial colonization on the implant surface by the formation of biofilm, which hinders the action of the immune system and antibiotics.^12^ ^,^ ^19^ ^,^ ^22^ ^,^ ^23^ ^,^ ^24^

The preoperative period and length of hospitalization have been identified as relevant factors for monitoring. A prolonged stay in the hospital environment exposes the patient to colonization by the local microbiota, favoring the emergence of infections.^17^ ^,^ ^25^ For some authors, the preoperative hospitalization period is related to a higher incidence of SSIs;^6^ ^,^ ^17^ ^,^ ^25^ the Anvisa recommendation,^2^ ^,^ ^6^ adopted by INTO, states that the time between admission and surgery does not exceed one day. The observed variability can be explained by the assistance to patients who reside in other states of the federation and who have been recommended high-complexity surgeries, thus remaining in the hospital while waiting for the procedure to be performed.

In this study, the total length of hospitalization was longer than that observed in adults.^25^ Kirkland et al.^22^ estimated the average time of total hospitalization at 11 days for cases and six days for control cases. Greene^26^ found that the incidence of SSIs in orthopedic surgeries increases the length of hospitalization by at least two weeks. This study, conducted with a pediatric population, established a difference of 23.8 days (3.4 weeks) between cases and control cases for the length of hospitalization, which is longer than that described for adults in the literature.

The multivariate analysis of patient characteristics and procedures revealed unidentified correlations in the bivariate analysis, such as association between age, weight, and weight Z-score and the incidence of SSIs, corroborating the findings of Glotzbecker et al.^20^

In the sample, we identified the record of a single patient who underwent spine surgery and developed an infection. He presented the four variables statistically related to the incidence of SSIs in his medical records. This patient was 36 months old at the time of surgery, remained hospitalized for 22 days until the day of surgery, used a total of 20 implants and was discharged 45 days after admission. In addition, he underwent thoracolumbar arthrodesis, combined with the lumbar hemivertebrectomy of seven vertebral segments, justifying his long recovery. The SSI diagnosis was made 66 days after surgery; the methicillin-sensitive *S. aureus* was isolated in the bone harvesting. The patient was malnourished (weight Z-score of -1.79), presented comorbidity (asthma), and the surgery lasted eight hours. All these variables are postulated as possible risk factors for the incidence of SSI and were identified in the correspondence analysis. It is well-known that the risk of SSI increases in any spinal surgical procedure, with rates varying between 3.7 and 8.5% depending on the population and the several factors involved in performing a surgery.^26^ ^,^ ^27^

Regarding follow-up data, the literature shows that the superficial incisional infection is the most common SSI.^9^ ^,^ ^17^ This data is compatible with the data found in the study. *S. aureus* was the most common etiologic agent, and one patient presented a MRSA profile, establishing the importance of this etiologic agent in the event of SSI.^9^ ^,^ ^17^ All patients with the confirmed agent had an implant. This clarifies the ability of the involved etiologic agents to form biofilm and supports the risk of SSI in the presence of implants.^24^

The average time to diagnosis of SSI was 26.5 days, ranging from 5 to 343 days. This can be explained by the fact that in most SSI cases the patient had a record of implant use, and therefore the infection may manifest later. In the sample, two cases were diagnosed in-hospital and remained hospitalized for the treatment of infection, which probably increased the average length of hospitalization. Currently, follow-up for patients with implants is 365 days. Nevertheless, different authors argue that SSIs manifest within a 21-day period, regardless of implants.^9^ ^,^ ^25^ ^,^ ^27^

One of the obstacles to precisely defining the SSI rate is the follow-up with patients after discharge. There are reports that SSI rates may be underestimated, since there is no specific protocol established by surveillance agencies for following up with discharged patients.^9^ ^,^ ^25^ ^,^ ^27^ This factor should be considered as these patients present an average of five outpatient visits, varying from 0 to 12 visits, up to one year after surgery. Compared to control groups, the number of visits did not reveal statistical significance; the average number of visits did not vary as well, suggesting that all patients had equal diagnostic opportunities.

Therefore, this study assessed the prevalence of SSIs in pediatric patients who underwent orthopedic surgery and identified variables (weight, age, weight Z-score, preoperative period, and length of hospitalization) that were related to their incidence. These variables should be considered in the process of SSI epidemiological surveillance for the pediatric population. There is a need for further research to verify the prevalence of post-surgical infections in the pediatric population. Investigations to estimate low-prevalence events should preferably be conducted in centers of excellence that concentrate more complex cases and specialists. Another valuable characteristic for conducting such studies is the availability of systematized data on hospitalizations and, most importantly, patient follow-up data.
